# eIF4E-Dependent Translational Control: A Central Mechanism for Regulation of Pain Plasticity

**DOI:** 10.3389/fgene.2018.00470

**Published:** 2018-10-24

**Authors:** Sonali Uttam, Calvin Wong, Theodore J. Price, Arkady Khoutorsky

**Affiliations:** ^1^Department of Anesthesia, McGill University, Montreal, QC, Canada; ^2^School of Behavioral and Brain Sciences, The University of Texas at Dallas, Richardson, TX, United States; ^3^Center for Advanced Pain Studies, The University of Texas at Dallas, Richardson, TX, United States; ^4^Alan Edwards Centre for Research on Pain, McGill University, Montreal, QC, Canada

**Keywords:** eIF4E, mRNA translation, persistent pain, sensitization, treatment

## Abstract

Translational control of gene expression has emerged as a key mechanism in regulating different forms of long-lasting neuronal plasticity. Maladaptive plastic reorganization of peripheral and spinal nociceptive circuits underlies many chronic pain states and relies on new gene expression. Accordingly, downregulation of mRNA translation in primary afferents and spinal dorsal horn neurons inhibits tissue injury-induced sensitization of nociceptive pathways, supporting a central role for translation dysregulation in the development of persistent pain. Translation is primarily regulated at the initiation stage via the coordinated activity of translation initiation factors. The mRNA cap-binding protein, eukaryotic translation initiation factor 4E (eIF4E), is involved in the recruitment of the ribosome to the mRNA cap structure, playing a central role in the regulation of translation initiation. eIF4E integrates inputs from the mTOR and ERK signaling pathways, both of which are activated in numerous painful conditions to regulate the translation of a subset of mRNAs. Many of these mRNAs are involved in the control of cell growth, proliferation, and neuroplasticity. However, the full repertoire of eIF4E-dependent mRNAs in the nervous system and their translation regulatory mechanisms remain largely unknown. In this review, we summarize the current evidence for the role of eIF4E-dependent translational control in the sensitization of pain circuits and present pharmacological approaches to target these mechanisms. Understanding eIF4E-dependent translational control mechanisms and their roles in aberrant plasticity of nociceptive circuits might reveal novel therapeutic targets to treat persistent pain states.

## Introduction

Chronic pain is a debilitating condition affecting more than 20 percent of the population worldwide (Steglitz et al., 2012; de Souza et al., 2017). Chronic pain is most commonly triggered by tissue inflammation or nerve injury, which can be caused by metabolic diseases (diabetes), autoimmune diseases, viral infection (herpes zoster), cancer, chemotherapy drugs (e.g., platinums, taxanes, epothilones, and vinca alkaloids), and nerve entrapment or blunt trauma. Chronic pain, however, can also appear without any recognizable trigger such as in fibromyalgia, migraine, irritable bowel syndrome, and interstitial cystitis.

In most cases, the pain is a result of increased sensitivity of peripheral or central nociceptive circuits to stimulation, causing painful sensation in response to a normally innocuous stimulus. The increase in sensitivity, also referred as sensitization, is mediated by a combination of mechanisms taking place at several levels along the pain pathway including primary sensory neurons, spinal cord, and higher brain areas (Todd, 2010; Yekkirala et al., 2017).

Long-lasting increases in the sensitivity and responsiveness of pain circuits is ultimately accompanied by changes in gene expression, which support biochemical and structural alterations in neuronal and non-neuronal cells involved in pain processing. Gene expression is a multi-step process that is tightly regulated at different levels. Regulation of the rate by which mRNA is translated into protein is called translational control (Sonenberg and Hinnebusch, 2009; Robichaud et al., 2018). Translational control has a strong impact on the abundance of proteins in the cell, and its dysregulation contributes to many pathologies in the nervous system including developmental abnormalities, metabolic dysregulation, autism spectrum disorder (ASD), and epilepsy (Buffington et al., 2014; Tahmasebi et al., 2018). Tissue injury, metabolic diseases, and certain drugs (e.g., anticancer and opioids) cause an upregulation of mRNA translation in pain-processing tissues such as dorsal root ganglion (DRG) and dorsal horn of the spinal cord (Melemedjian and Khoutorsky, 2015; Khoutorsky and Price, 2018). Inhibition of translational control signaling in these tissues reduces the sensitization of nociceptive circuits and alleviates pain, demonstrating a central role of translational upregulation in the development of persistent pain (Price et al., 2007; Jimenez-Diaz et al., 2008; Asante et al., 2009; Geranton et al., 2009; Price and Geranton, 2009; Melemedjian et al., 2010; Xu et al., 2011; Bogen et al., 2012; Ferrari et al., 2013; Obara and Hunt, 2014). The rate of mRNA translation is controlled via several mechanisms (Costa-Mattioli et al., 2009; Robichaud et al., 2018). The recruitment of the ribosome to the mRNA is a central step in translation initiation and the major site for regulation. A key mechanism to regulate this process is controlling the activity of the eukaryotic translation initiation factor 4E (eIF4E), which binds a mRNA “cap” structure (a 7-methylguanosine linked to the first nucleotide at the 5′ end of all nuclear transcribed eukaryotic mRNAs) and initiates ribosome recruitment (Altmann et al., 1985; Sonenberg and Hinnebusch, 2009). In this review, we focus on the regulation of eIF4E-dependent mRNA translation initiation in nociceptive plasticity, highlighting a central role of this mechanism in the development of chronic pain.

## Translational Control Mechanisms

**FIGURE 1:**
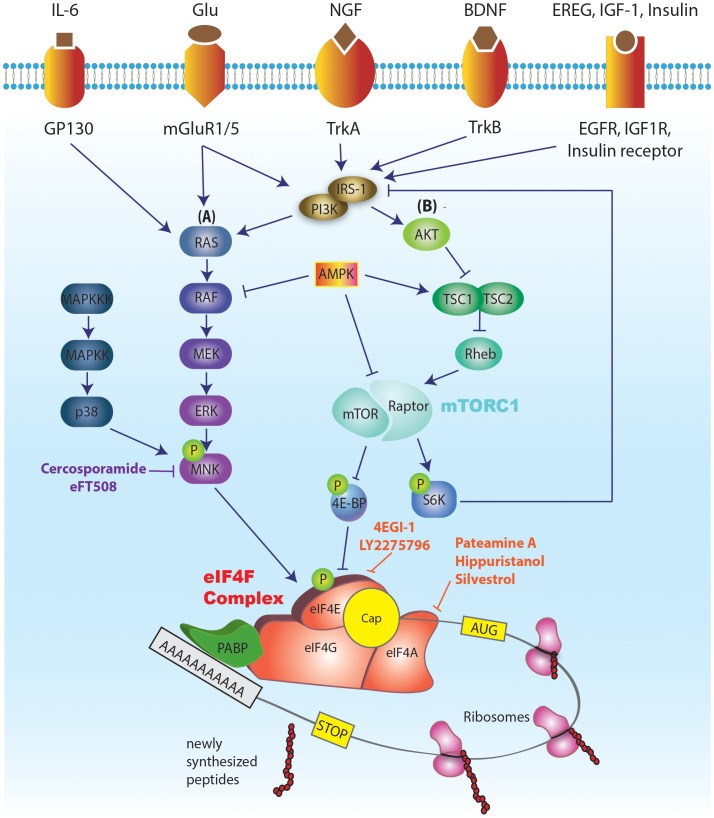
Schematic illustration of the major signaling pathways regulating eIF4E activity and translation initiation. The cap binding ability of eIF4E makes it a central regulator of translation. A critical step in the translation initiation process is the binding of eIF4E to the mRNA cap. eIF4E mediates the formation of the eIF4F complex on the mRNA cap structure (a 7mGp bound to the first nucleotide). eIF4F complex, in addition to eIF4E, consists of eIF4G (scaffolding protein) and eIF4A (helicase). Successful formation of eIF4F complex on the mRNA cap further promotes the recruitment of the pre-initiation complex (PIC), followed by 5′ UTR scanning to reach the start codon AUG and joining of 60S ribosomal subunit. This event marks the completion of translation initiation. eIF4E is a downstream effector of both mTORC1 (via 4E-BP-dependent repression) and ERK (via eIF4E phosphorylation by MNK 1/2). The activities of mTORC1 and ERK signaling pathways are in turn modulated by a multitude of external [tyrosine receptor kinase A (trkA) and trkB, receptors from the insulin receptor family (IR, IGF1R, EGFR), and metabotropic glutamate and NMDA receptors] and internal cues [status of cellular energy (via AMPK), oxygen levels (via activation of AMPK and REDD1; Regulated in DNA damage and development 1), and DNA damage (via the induction of p53 target genes)]. Various inhibitors of cap dependent translation initiation have been identified. 4EGI-1 inhibits eIF4E’s interaction with eIF4G, thus inhibiting the formation of eIF4F complex. Cercosporamide blocks MNK phosphorylation, which in turn prevents phosphorylation of eIF4E. Inhibitors of eIF4A have also been identified which function by either blocking its helicase activity (hippuristanol) or by preventing its participation in the eIF4F complex (pateamine A, and silvestrol).

The process of translation can be divided into three phases: initiation, elongation, and termination. Most of the regulation of translation occurs at the initiation step (Sonenberg and Hinnebusch, 2009; Merrick and Pavitt, 2018). Initiation is regulated by a large number of translation initiation factors, which mediate the recruitment of the ribosome to the mRNA, followed by scanning of the 5′ untranslated region (5′ UTR) of the mRNA for the presence of an AUG start codon. A critical step in this process is the binding of eIF4E to the mRNA cap. Following binding to the cap, eIF4E binds a mRNA helicase, eIF4A, and a large scaffolding protein, eIF4G, to form a tri-subunit complex named eIF4F (Figure 1). eIF4F facilitates the recruitment of the 43S preinitiation complex (PIC) to the mRNA. The PIC is composed of a small 40S ribosomal subunit, translation factors eIF1, eIF1A, and eIF3, and a ternary complex (eIF2: GTP bound to initiator, Met-tRNA_iMet_). Recruitment of the PIC is followed by scanning of the mRNA 5′ UTR and joining of a large ribosomal subunit (60S), upon encountering a start codon, to form an 80S ribosome that is competent to proceed to the elongation phase of translation. Importantly, the helicase activity of eIF4F (mediated by eIF4A) is required for unwinding the mRNAs 5′ UTR secondary structure to allow the scanning process and translation to proceed (Parsyan et al., 2011).

Other major mechanisms involved in the regulation of translation initiation include regulation of ternary complex availability [via phosphorylation of the alpha subunit of the eukaryotic initiation factor 2 (eIF2α)] (Trinh and Klann, 2013); regulation of the length of mRNA poly(A) tail which promotes translation and protects mRNA from degradation (Gray et al., 2000; Kahvejian et al., 2001; Derry et al., 2006); and finally translation initiation via a cap-independent mechanism (mediated by internal ribosome entry site, IRES) (Pelletier and Sonenberg, 1988; Macejak and Sarnow, 1991; Leppek et al., 2018). Since the expression levels of eIF4E are the lowest among all translation initiation factors, the formation of the eIF4F complex and correspondingly, translation initiation are the rate-limiting steps for translation under most circumstances.

## eIF4E is a Central Regulator of Cap-Dependent Translation

Eukaryotic translation initiation factor 4E activity is tightly regulated via two mechanisms. Translational repressor 4E-binding protein (4E-BP) binds eIF4E and prevents its association with eIF4G, and thus precludes the formation of the eIF4F complex (Gingras et al., 1999; Peter et al., 2015). In mammals, there are three 4E-BP isoforms – 4E-BP1, 4E-BP2, and 4E-BP3, which have similar functions but exhibit differences in tissue distribution. The binding of 4E-BP to eIF4E depends on the 4E-BP phosphorylation state. Upon phosphorylation by the mechanistic target of rapamycin complex 1 (mTORC1), the affinity of 4E-BP to eIF4E is reduced, leading to its dissociation from eIF4E and allowing the formation of eIF4F complex at the mRNA cap. This promotes the recruitment of 43S PIC to the mRNA and stimulation of translation (Figure 1).

Eukaryotic translation initiation factor 4E activity is required for translation initiation of all capped mRNAs. Complete loss of eIF4E, as in *eIF4E*^−/−^ mice is not compatible with life and leads to embryos death before embryonic day 6.5 (Truitt et al., 2015). Partial loss of eIF4E does not have a strong impact on general translation, mostly because it induces a compensatory degradation of hypophosphorylated 4E-BP1 (Yanagiya et al., 2012). Even though all nuclear transcribed eukaryotic mRNAs have a cap, not all cellular mRNAs are equally sensitive to eIF4E activity. The translation of “eIF4E-sensitive mRNAs” is preferentially stimulated by increased eIF4E activity. For example, housekeeping mRNAs such as *GAPDH* and β-actin are less sensitive to eIF4E as compared to mRNAs involved in cell growth, proliferation, and immune responses [e.g., c-MYC, cyclins, BCL-2, MCL1, osteopontin, survivin, vascular endothelial growth factor (VEGF), fibroblast growth factors (FGF), and matrix metalloproteinase 9 (MMP-9)] (Rousseau et al., 1996; Sonenberg and Gingras, 1998; Bhat et al., 2015; Chu and Pelletier, 2018). The mRNA features rendering eIF4E-sensitivity have been typically associated with 5′ UTRs enriched with high-complexity secondary structures (Pelletier and Sonenberg, 1985; Sonenberg and Gingras, 1998). It has been demonstrated that a long 5′ UTR favors the formation of stable secondary structures, and that the proximity of these structures to the cap obstructs eIF4F complex formation. On the other hand, hairpin structures with a greater free energy, located further away from the cap, restrict 5′ UTR scanning (the progression of the PIC toward the start codon) (Kozak, 1989; Pickering and Willis, 2005). However, translation of a subset of mRNAs without long 5′ UTR can still be sensitive to eIF4E, indicating that other 5′ UTR signatures may also render this sensitivity (Leppek et al., 2018). Potential mechanisms include the presence of 5′ terminal oligopyrimidine tracts (5′*TOPs*) (Thoreen et al., 2012) and *cis*-regulatory elements (Wolfe et al., 2014; Truitt et al., 2015; Hinnebusch et al., 2016; Truitt and Ruggero, 2016; Leppek et al., 2018) at the 5′ UTR. For example, a Cytosine-rich 15-nucleotide motif, termed Cytosine Enriched Regulator of Translation (CERT), was shown to be responsible for conferring eIF4E sensitivity under oncogenic transformation and oxidative stress (Truitt et al., 2015).

Although most studies have attributed the elevated translation of mRNAs with highly structured 5′ UTRs to the cap-binding ability of eIF4E and it being the limiting component of the eIF4F complex, other studies did not find that the cap-binding ability completely explained eIF4E function and explored further mechanisms of eIF4E-mediated translation regulation. This led to the identification of an additional function of eIF4E – stimulation of eIF4A helicase activity, which is independent of its cap-binding ability (Feoktistova et al., 2013). Feoktistova et al. (2013) showed that the eIF4E binding site on eIF4G has an autoinhibitory function. Binding of eIF4E to eIF4G counteracts this autoinhibition, and in turn enables eIF4G to stimulate eIF4A activity (rate of duplex unwinding). They show that this function of eIF4E is independent of its cap-binding activity, suggesting that eIF4E can stimulate translation by two distinct mechanisms (Feoktistova et al., 2013).

In addition to regulation by mTORC1/4E-BP, eIF4E activity is also controlled via phosphorylation of its sole phosphorylation site, Ser 209, by mitogen activated protein kinase [MAPK]-interacting protein kinases (MNKs) 1 and 2, downstream of the extracellular-signal-regulated kinase (ERK) and the p38 MAPK signaling cascades (Figure 1; Pyronnet et al., 1999; Waskiewicz et al., 1999). The phosphorylation of eIF4E is associated with altered translation of a subset of mRNAs, although the mechanisms underlying the effect of this phosphorylation event on translational efficiency and transcript-specificity remain elusive.

Since eIF4E is a downstream effector of both mTORC1 (via 4E-BP-dependent repression) and ERK (via eIF4E phosphorylation), its activity can be modulated by a multitude of external and internal cues that activate these central cellular signaling pathways. Numerous membrane receptors activate mTORC1 and ERK signaling in neurons including tyrosine receptor kinase A (trkA) and trkB, receptors from the insulin receptor family (IR, IGF1R, EGFR), and metabotropic glutamate and NMDA receptors. In addition to the extracellular cues, these pathways integrate intracellular signals conveying information on the status of cellular energy (via AMPK), oxygen levels [via activation of AMPK and REDD1 (Regulated in DNA damage and development 1)], and DNA damage (via the induction of p53 target genes) (Saxton and Sabatini, 2017; Figure 1).

## eIF4E in Regulation of Peripheral Nociceptive Plasticity

Tissue injury induces profound changes in the phenotype of sensory neurons, increasing their excitability and changing the connectivity within peripheral tissues and spinal cord. These alterations are driven by pro-inflammatory molecules released from injured tissues, such as neurotrophin nerve growth factor (NGF) and cytokine interleukin 6 (IL-6), as well as by neuronal activity evoked by direct injury to the nerve. ERK and mTORC1, two central intracellular pathways, are stimulated by tissue inflammation and nerve injury, diabetes, cancer, and drug-induced neuropathies (Melemedjian and Khoutorsky, 2015; Khoutorsky and Price, 2018). In addition to the phosphorylation-mediated activation of mTOR, downstream of PI3K/AKT pathway, a recent study showed that nerve injury stimulates local axonal *mTOR* mRNA translation (Terenzio et al., 2018). Translation profiling of DRG tissue from mice subjected to nerve injury showed that ERK is a key regulatory hub controlling both transcriptional and translation gene expression networks (Uttam et al., 2018).

Inhibition of ERK and mTORC1 signaling alleviates the development of pain hypersensitivity in a variety of pain models (Ji et al., 2009; Chen et al., 2018; Khoutorsky and Price, 2018). Since ERK and mTORC1 pathways converge on eIF4E to control the rate of cap-dependent translation, it was suggested that eIF4E might play a central role in the sensitization of pain circuits via regulating the translation of specific mRNAs. The physiological significance of eIF4E phosphorylation was studied using mice lacking eIF4E phosphorylation (knock-in mutation of serine^209^ to alanine, *eIF4E*^S209A^). These mice display greatly reduced mechanical and thermal hypersensitivity in response to intraplantar administration of IL-6, NGF, and carrageenan, as well as diminished hyperalgesic priming (Moy et al., 2017). Moreover, the increase in excitability of *eIF4E*^S209A^ primary sensory neurons in response to IL-6 and NGF was reduced as compared to wild-type (WT) controls. These findings were recapitulated in *MNK1/2* knockout mice, which also lack eIF4E phosphorylation. In the nerve injury model of neuropathic pain, spared nerve injury (SNI), the development of mechanical and cold hypersensitivity was reduced in both *eIF4E*^S209A^ and *MNK1/2* knockout mice. Notably, local intraplantar inhibition of MNK with cercosporamide reduced mechanical hypersensitivity in response to NGF and alleviated hyperalgesic priming (Moy et al., 2017). These findings support the notion that the stimulation of eIF4E phosphorylation is imperative for the phenotypic changes of sensory neurons, promoting the hyperalgesic state and contributing to the development of chronic pain, and that this likely occurs independently of effects on inflammation (Moy et al., 2018b). Experiments with local administration of cercosporamide also indicate that pro-inflammatory mediators- or tissue injury-induced phosphorylation of eIF4E mediates sensitization of sensory neurons via local mRNA translation.

The advances in translational profiling techniques have provided important insights into the potential mechanisms by which eIF4E phosphorylation regulates neuronal functions. In the brain, eIF4E phosphorylation controls the translation of mRNAs involved in inflammatory responses such as IκBα, a repressor of the transcription factor NF-κB that regulates the expression of the cytokine tumor necrosis factor (TNFα) (Aguilar-Valles et al., 2018). Genome-wide translational profiling of the brain from *eIF4E*^S209A^ mice revealed that eIF4E phosphorylation controls translation of mRNAs involved in inflammation (IL-2 and TNFα), organization of the extracellular matrix (*Prg2*, *Mmp9*, *Adamts16*, *Acan*), and the serotonin pathway (*Slc6a4*) (Amorim et al., 2018).

In the DRG, phosphorylation of eIF4E stimulates translation of brain derived neurotropic factor (*Bdnf*) mRNA. *eIF4E*^S209A^ mice show reduced protein levels of BDNF under baseline conditions and fail to translate *Bdnf* mRNA to protein in response to pro-inflammatory cytokines despite an increase in *Bdnf* mRNA levels (Moy et al., 2018a). BDNF is a key molecule mediating pain plasticity (Obata and Noguchi, 2006) and identification of MNK/eIF4E signaling as a central regulator of *Bdnf* translation has important therapeutic implications (Moy et al., 2018a). Cell-type specific translational profiling of nociceptors [using translating ribosome affinity purification (TRAP) approach] (Heiman et al., 2014) in a mouse model of chemotherapy-induced neuropathic pain revealed that MNK-eIF4E signaling controls translation of *RagA* mRNA, a key regulator of mTORC1 (Megat et al., 2018). This finding suggests crosstalk between ERK/MNK/eIF4E and mTORC1 signaling pathways in promoting pain hypersensitivity in chemotherapy-induced neuropathies.

In addition to phosphorylation, eIF4E in primary sensory neurons is also regulated via mTORC1/4E-BP. IL-6 and NGF activate mTORC1, which promotes 4E-BP1 phosphorylation, increased eIF4F complex formation and nascent protein synthesis in cultured sensory neurons (Melemedjian et al., 2010). Intraplantar administration of IL-6 or NGF induced mechanical allodynia, which is blocked by subcutaneous administration of the mTORC1 inhibitor rapamycin, as well as by 4EGI-1, an inhibitor of eIF4F complex formation that disrupts eIF4E and eIF4G interaction. Intraplantar 4EGI-1 also blocked the establishment of the sensitization state in a hyperalgesic priming model in response to IL-6 and NGF injection (Asiedu et al., 2011).

These findings support a model that local activation of mTORC1 stimulates eIF4F complex formation, promoting pain hypersensitivity via axonal mRNA translation. 4E-BP1 is a major isoform involved in regulation of nociception, whereas in the brain 4E-BP2 is the dominant isoform. 4E-BP1 is highly expressed in nociceptors and mice lacking 4E-BP1, but not 4E-BP2, exhibit enhanced mechanical hypersensitivity. Notably, *eif4ebp1* knockout mice show no alterations in thermal sensitivity, suggesting a mechanical-specific effect of eIF4E activation via 4E-BP-dependent mechanisms (Khoutorsky et al., 2015).

A second major downstream effector of mTORC1, p70S6 ribosomal kinase (S6K1 and S6K2) may not play as significant a role in the regulation of nociceptive sensitization. Mice lacking S6K1/2 do exhibit increased mechanical pain sensitivity, but normal thermal thresholds, and an inhibitor of S6K1/2 recapitulates this phenotype (Melemedjian et al., 2013). This finding seems paradoxical; however, further analysis revealed that loss of S6K1/2 function engages a feedback loop that stimulates enhanced ERK phosphorylation, driving mechanical sensitization (Melemedjian et al., 2013). Therefore, it is tempting to speculate that most of the pain inhibitory effects of mTORC1 inhibition are mediated via the suppression of 4E-BP1/eIF4E-dependent protein synthesis. The role of other translation-independent outputs of mTORC1, such as regulation of autophagy, lipogenesis, and mitochondrial function, remain unknown.

## eIF4E in Regulation of Spinal Plasticity

The spinal cord integrates peripheral somatosensory inputs to generate, after processing, an output that is conveyed to the brain where the perception of pain ultimately arises. Peripheral injury, disease, and certain drugs can cause an increase in the gain of spinal nociceptive circuits, resulting in disproportional amplification of somatosensory inputs, and therefore increased pain. These maladaptive plastic changes in the spinal cord, frequently referred to as central sensitization, significantly contribute to the development of pathological pain states. Central sensitization leads to a lowered threshold for the induction of pain (allodynia), an increase in the responsiveness to noxious stimuli (hyperalgesia), and an enlargement of the receptive field, resulting in pain sensation from non-injured areas (secondary hyperalgesia).

Long-lasting spinal plasticity critically relies on new protein synthesis to allow alterations in the cellular proteome, and consequently, sensitization of the pro-nociceptive circuits. Numerous studies have demonstrated the activation of ERK and mTORC1 signaling in the spinal cord following peripheral tissue injury, cancer, and opioid treatment (Geranton et al., 2009; Ji et al., 2009; Norsted Gregory et al., 2010; Xu et al., 2011, 2014; Shih et al., 2012; Jiang et al., 2013; Liang et al., 2013; Zhang et al., 2013). Intrathecal delivery of pharmacological inhibitors targeting these pathways efficiently alleviates pathological pain without affecting the baseline mechanical and thermal sensitivity (Ji et al., 2009; Melemedjian and Khoutorsky, 2015; Martin et al., 2017). There is evidence that the beneficial effect of mTORC1 inhibition on pain in the spinal cord is largely mediated via mTORC1/4E-BP1-dependent regulation of eIF4E activity. Pain hypersensitivity produced by intrathecal injection of epiregulin (EREG), an endogenous agonist of the epidermal growth factor receptor (EGFR) upstream of mTORC1, is blocked by intrathecal injection of 4EGI-1 (Martin et al., 2017). Moreover, specific deletion of 4E-BP1 in the dorsal horn of the spinal cord causes mechanical hypersensitivity (Khoutorsky et al., 2015). Mice lacking 4E-BP1 show increased excitatory and inhibitory synaptic transmission in lamina II neurons as well as enhanced potentiation of spinal excitatory field potentials following sciatic nerve stimulation. Taken together, these results indicate that enhanced eIF4F complex formation in the spinal cord promotes spinal plasticity and contributes to the development of central sensitization.

## Therapeutic Approaches to Target eIF4E-Dependent Mechanisms to Alleviate Pain

Several lines of evidence suggest that targeting eIF4E is a potentially promising therapeutic strategy to inhibit aberrant pain plasticity. First, due to low expression levels, eIF4E’s activity is a rate-limiting factor for translation initiation and a central node of regulation. eIF4E integrates signals from two major signaling pathways, ERK and mTORC1, both of which have important functions in the development of pain. Second, eIF4E does not strongly affect general translation, but mainly regulates the translation of a subset of mRNAs involved in cell growth, proliferation, immune responses, and neuronal plasticity. Mice with partial reduction of eIF4E protein levels, such as eIF4E heterozygous mice (Truitt et al., 2015) or mice expressing short hairpin RNA against eIF4E (Lin et al., 2012) show no developmental abnormalities or changes in survival rate or body weight. Third, whereas acute inhibition of mTORC1 is effective in alleviating pain, long-term mTORC1 inhibition leads to the hyperactivation of ERK via a mTORC1-S6K1-IRS1 negative feedback loop (Veilleux et al., 2010; Melemedjian et al., 2013). Since ERK is a well-known sensitizer of neurons involved in pain transmission, both in the periphery and the spinal cord, chronic mTORC1 inhibition leads to mechanical hypersensitivity and pain. Thus, long-term treatment with compounds targeting mTORC1 is unlikely to be clinically applicable. Conversely, chronic inhibition of eIF4E does not activate these compensatory mechanisms. Mice lacking eIF4E phosphorylation do not exhibit alterations in pain sensation at baseline, but show reduced nociceptive plasticity in response to pro-inflammatory and nerve injury stimuli (Moy et al., 2017). Finally, compelling preclinical studies have demonstrated beneficial effects of pharmacologically targeting eIF4E in alleviating persistent pain using 4EGI-1, an inhibitor of eIF4 complex formation or cercosporamide, an inhibitor of MNK. Efforts to develop and test new translation inhibitors are fuelled by their potential use for treatment of cancer (Stumpf and Ruggero, 2011), malaria (Baragana et al., 2015), and bacterial infection (Bhat et al., 2015). Here, we overview the existing and newly developed pharmacological approaches to target eIF4E-dependent translation.

### MNK Inhibitors

CGP57380 and cercosporamide are two small molecule inhibitors targeting MNK1 and MNK2 (Bhat et al., 2015). Cercosporamide, extracted from the fungus *Cercosporidium henningsii*, is an antifungal agent and a phytotoxin. It has antiproliferative and proapoptotic activities in cancer cells in preclinical animal models of lung and colon carcinomas (Konicek et al., 2011). It readily crosses the blood-brain barrier (BBB) and efficiently reduces p-eIF4E in the brain after peripheral administration (Gkogkas et al., 2013). However, both CGP57380 and cercosporamide have been shown to exhibit off-target effects (Bain et al., 2007; Bhat et al., 2015). More specific MNK inhibitors have been recently developed. eFT508 is a new generation Mnk1/2 inhibitor, which is potent, selective and orally bioavailable (Dreas et al., 2017). Its efficacy has been assessed in preclinical models of diffuse large B-cell lymphoma, and it causes a dose dependent decrease in eIF4E-phosphorylation (Reich et al., 2018). eFT508 is now in phase II clinical trial for the treatment of colorectal cancer. A recent study showed that eFT508 efficiently reduces eIF4E phosphorylation in DRG without affecting other major signaling pathways (ERK, 4E-BP, and AKT) and general translation (Megat et al., 2018). eFT508 also alleviated paclitaxel-induced mechanical and thermal sensitivity, supporting its further testing in other chronic pain conditions. BAY 1143269 is another potent, and selective orally administered MNK1 inhibitor (Santag et al., 2017). Additional MNK inhibitors include: 5-(2-(phenylamino)pyrimidin-4-yl)thiazol-2(3H)-one derivatives (Diab et al., 2014), resorcylic acid lactone analogs (Xu et al., 2013), and retinoic acid metabolism blocking agents (RAMBAs) (Ramalingam et al., 2014). These compounds need to be better characterized in both *in vitro* and *in vivo* studies.

### Inhibitors of eIF4F Complex

Three inhibitors disrupting eIF4G:eIF4E interaction have been described: 4EGI-1 (Moerke et al., 2007), 4E1RCat, and 4E2RCat (Cencic et al., 2011). 4EGI-1 is a small molecule, which binds eIF4E at the site distal to the eIF4G-binding epitope, causing localized conformational changes and dissociation of eIF4G from eIF4E (Papadopoulos et al., 2014). 4EGI-1 also impairs mitochondrial functions (Yang et al., 2015). 4EGI-1 has been used in studies examining the role of eIF4F complex in memory (Hoeffer et al., 2011) and autism (Gkogkas et al., 2013; Santini et al., 2013), where it was delivered directly to the brain (intracerebroventricular injection) as it does not readily penetrate the BBB. Rigidified analogs of 4EGI-1 have been developed, showing improved potency in inhibition of eIF4E/eIF4G interaction (Mahalingam et al., 2014).

4E1RCat, and 4E2RCat block the interaction of eIF4E with both eIF4G and 4E-BP1, and thereby prevent the eIF4F complex formation (Cencic et al., 2011). These compounds have not been used yet in the nervous system *in vivo*. Antisense oligonucleotide (ASO) targeting eIF4E (LY2275796) with improved tissue stability and nuclease resistance has been developed (Graff et al., 2007). Since eIF4E is overexpressed in many human cancers (by ∼3- to 10-fold) (Bhat et al., 2015), LY2275796 has been tested as an anti-cancer treatment. Administration of LY2275796 to patients resulted in a reduction of eIF4E mRNA and protein levels in tumor cells but caused dose-dependent toxicity (Hong et al., 2011). The antiviral drug ribavirin has been proposed to mimic the mRNA “cap” to inhibit eIF4E/mRNA interaction (Kentsis et al., 2004). This notion was later disputed, and ribavirin’s biological effects were attributed to translation-independent activities (Westman et al., 2005; Yan et al., 2005).

### eIF4A Inhibitors

eIF4A helicase activity is critically required for the eIF4F complex formation and unwinding of the 5′ UTR to allow scanning to occur. Therefore, targeting eIF4A might be an additional approach to inhibit eIF4F-dependent translation initiation, particularly for mRNAs with highly structured 5′ UTRs. Pateamine A, hippuristanol, and recoglate family members [e.g., silvestrol and Rocaglamide A (RocA)] are the commonly known inhibitors of eIF4A, out of which only pateamine A is known to cause irreversible inhibition (Pelletier et al., 2015). Hippuristanol is a member of the polyoxygenated steroids family, and it blocks the helicase activity of eIF4A by binding to the C-terminal of eIF4A and imposing allosteric hindrance, thus preventing eIF4A to bind RNA (Sun et al., 2014). On the other hand, pateamine A increases the sequence non-specific RNA-binding activity of free eIF4A, thus preventing eIF4A from participating in the formation of eIF4 complex (Bordeleau et al., 2006; Cencic et al., 2009). Out of these eIF4A inhibitors, silvestrol has been most widely assessed in *in vivo* preclinical cancer models, owing to its high potency, bioavailability, and relatively low toxicity (Raynaud et al., 2007). Recently, RocA was identified as a sequence-selective inhibitor of translation which acts by stabilizing eIF4A binding on polyurine sequences, thus impeding 43S scanning and leading to upstream premature translation initiation (Iwasaki et al., 2016). The anticancer potential of rocaglates has been widely examined, however, the mechanisms underlying their cytotoxic and anti-proliferative effects have been studied only recently (Becker et al., 2016). The role of eIF4A inhibitors in pain has yet to be examined.

## Conclusion

A central role of eIF4E-dependent translational control in mediating maladaptive nociceptive plasticity provides an opportunity to develop new therapeutics to prevent the development of the hypersensitivity state or even reverse established pain states by weakening ongoing activity-dependent plasticity. Existing compounds targeting eIF4E (cercosporamide and 4EGI-1) lack specificity and have poor solubility and BBB permeability (4EGI-1). Therefore, validation of other existing inhibitors for *in vivo* applications and development of more specific and efficacious inhibitors are required. Another important research direction is uncovering cell type-specific translational landscapes (for example using TRAP) in different pain conditions. This work might reveal mRNAs whose aberrant translation drives the pain phenotype and allow targeting these transcripts or the encoded proteins to reverse the hypersensitivity. It is, however, conceivable that a complex pattern of translation drives the hypersensitivity, involving a combinatory effect of several translationally activated and repressed mRNAs. In this scenario, targeting upstream regulatory mechanisms, such as formation of eIF4F complex, might be a more feasible therapeutic approach. Combination of diverse inhibition strategies could be beneficial to achieve long-lasting effects on pain without triggering compensatory mechanisms.

In summary, a growing recognition of the importance of the eIF4E-dependent translational control in regulation of cellular functions in general and neuronal plasticity in particular, have substantially accelerated studies in the field of pain and advanced our knowledge of how eIF4E-dependent translational dysregulation causes maladaptive plasticity and contributes to the sensitization of the pain pathway. Identification of new molecular targets and pharmacological compounds to target these mechanisms might constitute a basis for next-generation pain therapeutics.

## Author Contributions

All authors participated in writing the manuscript.

## Conflict of Interest Statement

The authors declare that the research was conducted in the absence of any commercial or financial relationships that could be construed as a potential conflict of interest.
